# Modifying Our Environment to Improve Outcomes in Inflammatory Bowel Diseases?

**DOI:** 10.1002/ueg2.12734

**Published:** 2024-12-12

**Authors:** Mathurin Fumery, Nicolas Richard

**Affiliations:** ^1^ Department of Gastroenterology CHU Amiens and PériTox UMR‐I 01 INERIS Picardie Jules Verne University Amiens France; ^2^ Department of Gastroenterology University Rouen Normandie INSERM, ADEN UMR1073 “Nutrition, Inflammation and Microbiota‐Gut‐Brain Axis” CHU Rouen Rouen France

**Keywords:** Crohn's disease, environemental factors, inflammatory bowel disease, population‐based, smoking

Over the past decades, inflammatory bowel disease (IBD) has emerged as a significant public health challenge worldwide. In Europe alone, it is estimated that around 1.3 million people suffer from IBD, representing at least 0.2% of the population [1, 2]. Numerous environmental factors—such as smoking, urban living, consumption of ultra‐processed food, and exposure to antibiotics—have been linked to an increased risk of developing IBD, with varying levels of evidence [3]. Developing a robust understanding of how modifiable environmental exposures impact IBD risk is essential for informing effective prevention strategies. Similarly, identifying modifiable environmental factors that influence the disease course could guide interventions to mitigate risks and slow disease progression among IBD patients. While smoking and ultra‐processed foods have been associated with active disease or worse outcomes, there is a paucity of data on the broader impact of environmental factors on IBD activity, severity, and phenotype [4, 5].

In the current issue of the journal, Attauabi et al. present the first prospective population‐based study examining the impact of various environmental factors—collected at the time of diagnosis—on the clinical presentation and short‐term disease course of early IBD [6]. The study utilized data from the well‐established prospective Copenhagen IBD Inception Cohort, which included 208 and 128 incident UC and CD patients, respectively, diagnosed between 2021 and 2023. This cohort was meticulously monitored using clinical activity indices (Simple Clinical Colitis Activity Index, Harvey‐Bradshaw Index), biomarkers (CRP, calprotectin), and endoscopic assessments (Mayo Endoscopic Score, Simple Endoscopic Score‐CD), enabling the evaluation of disease activity and complication. Data on environmental factors were captured through International Organization for the Study of IBD (IOIBD) and HeartDiet questionnaires, which encompass 87 questions covering 25 key areas of environmental risk. These included early‐life factors (e.g., breastfeeding, exposure to pets), dietary and physical activity habits prior to diagnosis, smoking status, sanitary conditions, and the use of specific medications such as contraceptives. Early disease course (3 months) was assessed, with severe disease course defined as the need for oral steroids, immunomodulators, biologics, or colectomy.

Despite the relatively small cohort, the authors made several notable observations. The adverse impact of smoking use was reaffirmed in CD, with smokers experiencing higher rates of hospitalizations and a greater likelihood of developing a stricturing phenotype. Conversely, certain healthy dietary behaviors—such as daily consumption of fruits and vegetables and a high fiber intake—were associated with a reduced occurrence of stricturing or penetrating CD phenotypes.

Of course, the limitations of this study must be acknowledged. These include the small sample size, short follow‐up period, and the absence of data on certain critical environmental risk factors. Future environmental studies will have to also consider the role of pollutants such as heavy metals, air pollutants, per‐ and polyfluoroalkyl substances (PFAS), and pesticides, which have been linked to an increased risk of IBD [7]. Their influence on disease activity and progression remains an important question for further research. Additionally, while the study highlighted that exposure to multiple environmental factors was associated with a younger age at diagnosis, the potential interaction effects between these factors require exploration in larger, well‐characterized cohorts. Future research should also account for the influence of genetic background and other modifiers. Moreover, analyses on the duration and timing of environmental exposures in relation to IBD outcomes are needed to propose relevant environmental and nutritional interventions for newly diagnosed IBD patients.

In conclusion, while awaiting high‐level evidence from further studies and interventional trials, the findings of Attauabi et al. underscore the importance of providing personalized guidance to newly diagnosed IBD patients. This includes smoking cessation and the promotion of healthy dietary habits, which could offer benefits far beyond intestinal health.

## Conflicts of Interest

M.F.: Consulting/lecture fees for Abbvie, Amgen, Arena, Biogen, Celtrion, CTMA, Galapagos, Janssen, MSD, Pfizer, Takeda, Tillotts. MSD, Gilead, Fresenius, Celgene, Sandoz and Ferring. N.R.: Consulting/lecture fees for Abbvie, Ferring, Janssen and Takeda.

## Data Availability

Data sharing not applicable to this article as no datasets were generated or analyzed during the current study.
